# Using Systolic Local Mechanical Load to Predict Fiber Orientation in Ventricles

**DOI:** 10.3389/fphys.2020.00467

**Published:** 2020-06-09

**Authors:** Takumi Washio, Seiryo Sugiura, Jun-ichi Okada, Toshiaki Hisada

**Affiliations:** ^1^UT-Heart Inc., Kashiwanoha Campus Satellite, Kashiwa, Japan; ^2^Future Center Initiative, Kashiwanoha Campus Satellite, University of Tokyo, Kashiwa, Japan

**Keywords:** fiber orientation, ventricle, active stress, branching structure, mechanical load, remodeling, infarction

## Abstract

A simple rule adopted for myofiber reorientation in the ventricles is pursued by taking the microscopic branching network of myocytes into account. The macroscopic active tension generated on the microscopic branching structure is modeled by a multidirectional active stress tensor, which is defined as a function of the strains in the branching directions. In our reorientation algorithm, the principal direction of the branching network is updated so that it turns in the direction of greater active tension in the isovolumetric systole. Updates are performed step-by-step after the mechanical equilibrium has been attained with the current fiber structure. Starting from a nearly flat distribution of the principal fiber orientation along the circumferential direction, the reoriented fiber helix angles range from 70 to 40° at epicardium and from 60 to 80° at endocardium, in agreement with experimental observations. The helical ventricular myocardial band of Torrent-Guasp’s model and the apical spiral structure of Rushmer’s model are also reconstructed by our algorithm. Applying our algorithm to the infarcted ventricle model, the fiber structure near the infarcted site is remodeled so that the helix angle becomes steeper with respect to the circumferential direction near the epicardial surface. Based on our numerical analysis, we draw the following conclusions. (i) The multidirectional active tension based on the microscopic branching network is potentially used to seek tighter connection with neighboring aggregates. (ii) The thickening and thinning transitions in response to active tension in each myocyte allow the macroscopic principal fiber orientation of the microscopic branching network to move toward the direction of greater active tension. (iii) The force–velocity relationship is the key factor in transferring the fiber shortening strain to the magnitude of active tensions used in the myofiber reorientation. (iv) The algorithm naturally leads to homogeneity in the macroscopic active tension and the fiber shortening strain, and results in near-optimal pumping performance. (v) However, the reorientation mechanism may degrade the pumping performance if there is severely inhomogeneous contractility resulting from infarction. Our goal is to provide a tool to predict the fiber architecture of various heart disease patients for numerical simulations of their treatment plans.

## Introduction

**FIGURE 1:**
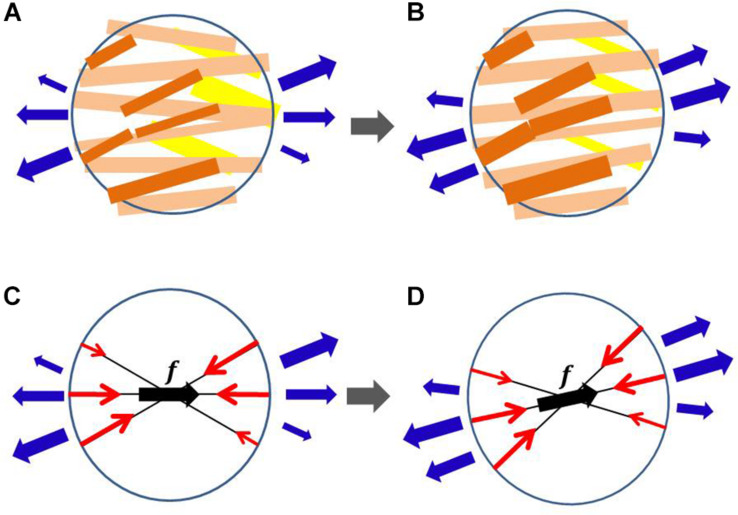
Myofiber reorientation process in the microscopic branching network of myocytes **(A,B)** and the associated macroscopic representation by the multidirectional branching active stress **(C,D)**. The myocytes with greater active tension are colored dark orange, whereas those with smaller active tension are colored yellow in **(A,B)**. The macroscopic representations of the multidirectional active stresses are given in **(C,D)**, in which ***f*** denotes the principal fiber direction, and the length of the red arrows represents the aggregated magnitude of active tension in each direction. The external tensions imposed by the neighboring aggregates are represented by blue arrows. In the reorientation process, we assume that the myocytes that sense greater tension in **(A)** become thicker in **(B)** and those that sense smaller tension in **(A)** become thinner in **(B)**. The microscopic thickening and thinning process is macroscopically modeled so that the eccentricity of active tensions from the principal fiber direction is diminished. The changes in external tensions (blue arrows) from **(A,C)** to **(B,D)** represent the reorientation in neighboring aggregates.

The distribution of fiber orientation in the ventricle wall is a key factor in determining the volume of blood ejected, local mechanical load, and energy consumption. Ideally, the fiber structure should maximize the ejection fraction while minimizing the mechanical load and energy consumption. However, such optimization is difficult to attain because of the complicated relationship between the active tension, energy consumption, and muscle deformation. In particular, the active tension is not determined by the strain alone, but also by the shortening velocity along the fiber orientation. Nevertheless, in our previous work (Washio et al., 2016) we showed that such optimization can be performed by sensing the multidirectional impulses in the microscopic branching myofibril structure during the systolic phase and shifting the center of the branches toward the direction of greatest impulse (Figure 1). Surprisingly, we found that this process constructed a realistic fiber structure of the left ventricle, starting from an almost flat distribution of fiber orientations along the circumferential direction. Furthermore, we saw that the distributions of active tension, sarcomere shortening velocity, and energy consumption were much more uniform than that of the artificial fiber structure with a linearly interpolated transmural helical fiber angle arrangement. The active tension decreases as the fiber shortening velocity increases (the force–velocity relationship, Hunter et al., 1998). Therefore, the strain and stress along the fiber orientation are strongly correlated. Our previous work suggests that the uniformity of the fiber shortening velocity could be naturally realized by examining the fiber structure in terms of the uniform active stress. In addition, the uniformity of the fiber active stress could be realized by using the microscopic branching fiber architecture. This macroscopic reorientation mechanism can be understood microscopically, as depicted in Figure 1. Under the force–velocity relationship in myofibrils, myocytes that are strongly pulled by their neighboring myocytes generate greater active tension, because they are shortened slower than other myocytes. If the myocytes that sense greater tension along their longitudinal direction become thicker, the principal fiber direction in the local aggregate can be reoriented to the new direction, strengthening the connections with the neighboring aggregates.

In this paper, to predict the fiber orientation in the ventricle, we propose a reorientation algorithm in which a simple active stress model that refers only to the strains in the multidirectional fibers around the principal fiber orientation is used instead of the sophisticated model that refers also to the fiber shortening velocities. In the simple model, the strains in the multidirectional fibers are referenced to compute the active stresses in the associated directions, and the obtained stresses are superposed to reproduce the macroscopic active stress on the branching network. Furthermore, the static equilibrium under isovolumetric condition is computed instead of the equilibrium in the whole dynamical systole. This contrasts with the sophisticated cross-bridge model used to compute dozens of heartbeats in our previous work. In the previous approach, the fiber structure was updated by referring to the active tension impulses in the branching directions after each heartbeat. Thus, dozens of heartbeats were needed to identify the final structure. In this study, we simulate only the isovolumetric contraction phase, and update the fiber structure step-by-step after finding the mechanical equilibrium for the updated fiber structure. Thus, the computational cost is considerably less than that of the previous approach. The reason for using the isovolumetric phase is that we can limit the range of overall fiber shortening strain because of the constraint on the cavity volume. Thus, the force–velocity relationship in the dynamic condition can be approximately simulated by a simple linear relationship between the active tension and the fiber shortening strain under small deformations in the static condition. This simplified active stress model highlights the essential factors involved in the myofiber reorientation mechanism.

Rijcken et al. (1997) showed that the physiological fiber helix angle can be predicted by minimizing the integrated variance of fiber stain at the beginning of the ejection stage. Their work was later extended to consider the whole ejection process (Rijcken et al., 1999). The design space of the fiber helix angle is limited to two parameters that characterize the linear function which approximates the transmural change in the fiber helix angle in the rotationally symmetric left ventricular model, whereas our reorientation process allows changes at each individual location in the three-dimensional biventricular model. Rijcken et al. considered an objective function given by integrating the variance of fiber strains, and optimized this function for three parameters that characterize the fiber structure; in contrast, we do not have any specific objective function, instead using only local information about the mechanical loads. Kroon et al. (2009) hypothesized that myofibers adapt their local orientation to achieve minimal fiber–cross-fiber shear strain during the cardiac cycle, and proposed a simple reorientation process that uses only the local deformation gradient tensor to update the fiber orientation. Their idea is based on breaking and forming connections between the extra-cellular matrix and myofibers, whereas our method is based on the thickening and thinning of myocytes in the microscopic branching network. In this paper, we show that our reorientation process attains a physiologically reasonable fiber structure, even when starting from a nearly flat distribution of fiber orientation along the circumferential direction; such a large reorientation of helix angles was not tested by Kroon et al. (2009) or in subsequent studies (Pluijmert et al., 2012, 2017).

Regarding the possibility of personalized fiber structure modeling, a quite different approach was proposed by Lekadir et al. (2014) who used statistical data to predict the fiber structure. This takes inter-subject variability in the ventricular shape into account, and estimates the fiber orientation at each individual myocardial location. Because our algorithm allows any initial fiber orientation distribution, and also permits inhomogeneous contractility, it can be used to modify the fiber structure obtained by an existing prediction tool toward a more mechanically reasonable form.

## Materials and Methods

### Active Stress of Multidirectional Fibers

We represent the configuration of the unloaded ventricle by Ω, and call this the reference configuration. The current position of the material point **X** ∈ Ω is represented as **x** = **x**(**X**) (see Supplementary Material S1 for Mathematical notations and the derivation of active stress tensor). The local infinitesimal deformation from the reference configuration is represented by the deformation gradient tensor **F** = ∂⁡**x**/∂⁡**X**. The active stress along the fiber direction, represented by the unit vector **f** in the reference configuration, is given by the second Piola–Kirchhoff stress tensor as

(1) $${\text{S}}_{a\,c\,t}=\frac{T_{f}}{||\mathrm{Ff}||}\,\text{f}⊗\text{f},$$

where *T*_*f*_ is the active tension (contraction force per unit area in the reference configuration). The above definition of the second Piola–Kirchhoff stress tensor means that the traction force **dt** in the current configuration acting on an area element **N***dA* in the reference configuration, in which the normal vector **N** points outward from the area, is given as follows:

(2) $$\text{dt}={\text{FS}}_{a\,c\,t}\,\text{N}\,d\,A=T_{f}\,\frac{\text{Ff}}{||\mathrm{Ff}||}\,\left(\text{f}\cdot \text{N}\right)\,d\,A,$$

where $\frac{\mathrm{Ff}}{||\mathrm{Ff}||}$ is the unit vector directed along the fiber orientation direction in the current configuration and (**f**⋅**N**) is proportional to the number of myofibrils that pass through the unit area perpendicular to **N**. This interpretation justifies the definition of **S**_*act*_. Note that **S**_*act*_**N***dA* itself represents **F**^−1^**dt** from the definition of the second Piola–Kirchhoff stress tensor.

To use the reorientation process based on the mechanical equilibrium condition in the steady state, we define the multidirectional active stress tensor **S**_*act*,*MD*_ of the branching fiber orientations ${\left({\text{f}}_{i}\right)}_{i=0}^{n}$ as

(3) $${\text{S}}_{a\,c\,t,M\,D}=T_{r\,e\,f}\,\sum_{i=0}^{n}\frac{w_{i}\,\Psi \,\left(\in_{i}\right)}{||{\text{Ff}}_{i}||}\,{\text{f}}_{i}⊗{\text{f}}_{i},$$

where *T*_*ref*_ is the reference value of the active tension and *w*_*i*_ is the weight assigned to the *i*-th direction. A value of *T*_*ref*_ = 40 kPa was adopted in our numerical experiments. In our pervious simulation work of beating ventricle (Washio et al., 2016) the active tension raised approximately up to 80 kPa. Therefore, we took the averaged value of active tension. The feedback from the deformation to the active stress is given by Ψ( ∈_*i*_), which is a function of the contraction ratio from the end diastolic configuration given by

(4) $$\in_{i}=1-\frac{||{\text{Ff}}_{i}||}{||{\text{F}}_{E\,D}\,{\text{f}}_{i}||},i=0,\ldots ,n,$$

where **F**_*ED*_ is the deformation gradient tensor at the end diastole. Ψ is defined such that the active tension decreases linearly to zero until the contraction ratio ∈ _*i*_ reaches ∈_*Z*_, i.e.,

(5) $$\Psi \,\left(\in_{i}\right)=\left\{ \begin{array}{cc} 1, & \in_{i}<0\\ 1-\in_{i}/\in_{Z,} & 0\leq \in_{i}\leq \in_{Z}\\ 0, & \in_{i},>,\in_{Z} \end{array}\right.$$

In the numerical experiments, ∈_*Z*_ was set to 0.1 unless specified. As ∈_*Z*_ becomes smaller, the sensitivity of the stress to the strain increases. The function Ψ is defined so that it approximates the force–velocity relationship under the dynamic condition by the stress–strain relationship under the static condition. We do not focus on the actual magnitude of force and velocity, but rather the response of the force to small changes in velocity. Later, the validity of the simplification from the dynamic condition to the static condition will be confirmed by the insensitivity of the reoriented fiber structures to the parameters *T*_*ref*_ and ∈_*Z*_. Ψ is set to be one in the case of stretching ( ∈ _*i*_ < 0) because the increase of active stress due to stretching may mislead the reorientation.

The branching fiber orientations are based on the orthonormal frame {**f**,**s**,**n**} as follows:

(6) $${\text{f}}_{0}=\text{f},$$

(7) $${\text{f}}_{i}=\cos\theta \,\text{f}+\sin\theta \,\left(\cos\frac{2\,\pi \,\left(i-1\right)}{n}\,\text{s}+\sin\frac{2\,\pi \,\left(i-1\right)}{n}\,\text{n}\right),$$

where **s** is the sheet orientation vector, which usually indicates the laminar plane together with the principal fiber orientation **f**. However, the orientation of **s** is not essential in this study, because the sheet direction is only used to determine the branching directions in Eq. (7). The parameter θ is associated with the dispersion degree of the branching structure. In the numerical experiments, the branching angle θ was set to 45° unless specified, and the weights {*w*_*i*_} were determined for the principal orientation as *w*_0_ = 1/2 and for the eight peripheral orientations as *w*_*i*_ = 1/16(1≤*i*≤8). Note that these parameters do not necessarily reflect the physiological microscopic structure of the branching network, because our purpose is not to predict the pumping performance, but to predict the fiber structure. As we will see in the numerical experiments, the parameters have little effect on the obtained fiber structure.

### Mechanical Equilibrium Condition

In the proposed method, the fiber structure is updated under the mechanical equilibrium in the isovolumetric contraction phase. The equilibrium equation is represented as follows:

(8) $$\int_{\Omega }\delta \,\text{E}:\left({\text{S}}_{a\,c\,t,M\,D}+{\text{S}}_{p\,a\,s}+2\,p\,J\,\text{I}\right)\,d\,\Omega =P_{L}\,\int_{\Gamma \,L}\delta \,\text{u}\cdot {\text{n}}_{\Gamma }\cdot d\Gamma_{L}+P_{R}\,\int_{\Gamma \,R}\delta \,\text{u}\cdot {\text{n}}_{\Gamma }\cdot d\Gamma_{R},$$

(9) $$\int_{\Omega }\delta \,p\,\left(2\,\left(J-1\right)-\frac{p}{k}\right)\,d\Omega =0,$$

(10) $$\left\{ \begin{array}{cc} V_{L}= & V_{L,E\,D}\\ V_{R}= & V_{R,E\,D} \end{array}\right.,$$

where **u** = **u**(**X**) = **x**(**X**)−**X** is the displacement of material point **X** ∈ Ω, $\text{E}=\frac{1}{2}\,\left({\text{F}}^{T}\,\text{F}\text{ –}\text{I}\right)$ is the Green–Lagrange strain tensor, *J* = det⁡**F** is the Jacobian, *p* is the hydrostatic pressure, k = 200 kPa is the bulk modulus, and *P*_*L*_ and *P*_*R*_ are the blood pressures in the left and right ventricles, respectively. *Γ*_*L*_ and Γ_*R*_ are the intracavity boundaries of the left and right ventricles, respectively, in the current configuration, and *n*_Γ_ is the outward normal unit vector from these surfaces. The Dirichlet boundary condition **u**(**X**) = 0 is imposed on the boundary nodes around the valve rings. The last condition is the isovolumetric constraint, in which *V*_*L*,*ED*_ and *V*_*R*,*ED*_ are the cavity volumes, respectively, of the left and right ventricles at the end diastole. **S**_*pas*_ is the passive second Piola–Kirchhoff stress tensor, defined from the deformation potential *W* as

(11) $${\text{S}}_{p\,a\,s}=\frac{\partial W}{\partial \text{E}}$$

In this study, the deformation potential *W* is given by

(12) $$W=c_{1}\,\left({\tilde{I}}_{1}-3\right)+c_{2}\,\frac{\exp\left(q_{2}\,\text{E}:\text{E}\right)-1}{2}$$

Here, ${\tilde{I}}_{1}$ is the reduced invariant, defined as

(13) $${\tilde{I}}_{1}=\det{\left(C\right)}^{-\frac{1}{s}}\,T\,r\,\left(C\right)$$

with the right Cauchy–Green deformation tensor **C**=**F**^*T*^**F**. We set *c*_1_ = 25 Pa, *c*_2_ = 800 Pa, and *q*_2_ = 4. Applying the second term alone is unstable for small deformations, and so the first term was added. Because our focus is the impact of the fiber structure on the active stress, we did not introduce the anisotropy in the passive deformation energy caused by the fiber–laminar structure, which is specified by the distribution of fiber-sheet vectors (Usyk et al., 2000).

### Myofiber Reorientation Algorithm

Given the initial frame distribution {**f**,**s**,**n**}, the internal left and right ventricle cavity pressures are linearly increased up to the prescribed end diastolic pressures *P*_*L*,*ED*_ and *P*_*R*,*ED*_, respectively. In our simulations, values of *P*_*L*,*ED*_ = 12 mmHg and *P*_*R*,*ED*_ = 4 mmHg were adopted. During the expansion process of the cavity volumes, the active stress tensor is set to zero. Once the mechanical equilibrium at the end diastole has been established, the stretching along the branching directions ||**F**_*ED*_**f**_*i*_|| is recorded for all elements in the ventricle. The mechanical equilibrium is then computed in the isovolumetric contraction phase, where the reference value *T*_*ref*_ of active stress is gradually increased to the prescribed reference value. During this process, the cavity volumes of the left and right ventricles are constrained to the end diastolic volumes, *V*_*L,ED*_ and *V*_*R*,*ED*_, respectively. Once the mechanical equilibrium has been attained with the prescribed reference value *T*_*ref*_, the reorientation process starts. In this process, the frames {**f**,**s**,**n**} of all elements are updated by the following procedure. First, the candidate new principal direction $\text{e}=\tilde{\text{e}}/||\tilde{\text{e}}||$ of the multidirectional active tensor **S**_*act*,*MD*_ in Eq. (3) is computed by summing the weighted vectors:

(14) $$\tilde{\text{e}}=\sum_{i=0}^{n}w_{i}\,\Psi \,\left(\in_{i}\right)\,{\text{f}}_{i}.$$

The angle $\tilde{\eta }$ between the principal fiber orientation vector **f** and the vector **e** is then computed as

(15) $$\cos\tilde{\eta }=\text{f}\cdot \text{e}.$$

As the rotation angle from **f** to **e** in determining the new fiber orientation, a small angle is taken by multiplying the weight ω by $\tilde{\eta }$:

(16) $$\eta =\omega \,\tilde{\eta },$$

because the application of $\tilde{\eta }$ itself to the rotation of the frame {**f**,**s**,**n**} may cause the Newton iterations to break down while computing the mechanical equilibrium after updating the frame. In this study, a value of ω = 0.05 was adopted. The axis of rotation **r**_f_ is determined so that it is perpendicular to **f** and **e**. Namely, the unit vector $\text{g}=\tilde{\text{g}}/||\tilde{\text{g}}||$ is computed from $\tilde{\text{g}}=\text{e}-\left(\text{e}\cdot \text{f}\right)\,\text{f}$, and then the axis is computed from **r**_f_ = **f**×**g**. The rotation **R**(η), represented as

(17) $$\text{R}\,\left(\eta \right)=\left[\begin{array}{ccc} \cos\eta & -\sin\eta & 0\\ \sin\eta & \cos\eta & 0\\ 0 & 0 & 1 \end{array}\right],$$

with respect to the orthonormal basis {**f**,**g**,**r**_f_} is applied to update each basis in the frame {**f**,**s**,**n**}. After updating the frame, the stretch ratios along the branching directions {||**F**_*ED*_**f**_*i*_||} in Eq. (4) are recomputed for the end diastolic deformation. The mechanical equilibrium is computed again for the next update. It is not clear whether there is a stationary fiber structure in this reorientation process. Though we expect a monotonic increase in the intracavitary pressures *P*_*L*_ and *P*_*R*_, it is possible that they could decrease during the reorientation process. To avoid such a case, we also introduce a modified reorientation process that is insensitive to small eccentricity in the vector $\tilde{\text{e}}$ in Eq. (14). Namely, we introduce a threshold value ${\tilde{\eta }}_{T}$, and do not update the frame if the following condition is satisfied:

(18) $$\tilde{\eta }\leq {\tilde{\eta }}_{T}.$$

Using this strategy to ensure insensitivity to small eccentricity, we could expect the evolution of the fiber structure to stop at some point. In the numerical experiments, this insensitivity mechanism was applied with the threshold ${\tilde{\eta }}_{T}=1^{\circ }$ unless otherwise specified.

### Finite Element Ventricular Model and Computation Resource

In the finite element modeling, the ventricular walls were discretized with 45,000 tetrahedral elements, where the MINI(5/4c) element (Brezzi and Fortin, 1991) was adopted to avoid the instability caused by the nearly incompressible condition in Eq. (9). Though the higher-order interpolation of MINI elements was applied to the displacement **u** to evaluate the integration associated with the passive stress tensor, standard linear interpolation ignoring the central node was adopted for the active stress. Thus, it was sufficient to assign one basis {**f**,**s**,**n**} for each element. Therefore, reorientation based on Eq. (14) was performed by referencing the contraction ratio ∈_*i*_, which was computed at the central integration point of each tetrahedral element. The computations were performed using a parallel computer system (Intel Xeon E-2670 [2.6 GHz], 16 cores; Intel, Santa Clara, CA, United States) with a computation time of 14 s for a single update.

## Results

### Evolution of Fiber Structure From a Nearly No-Twisting Structure

**FIGURE 2:**
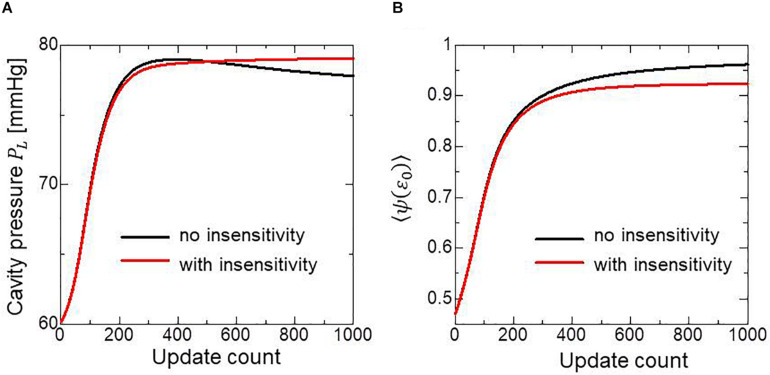
Changes in the left ventricular intracavitary pressure **(A)** and the averaged active stress factors in the principal fiber direction **(B)** during the reorientation process with (red lines) and without (black lines) the insensitivity mechanism. When the insensitivity mechanism was not adopted, the intracavitary pressure gently decreased after reaching the maximum (**A:** black line), though the active stress factor monotonically increased (**B:** black line).

**FIGURE 3:**
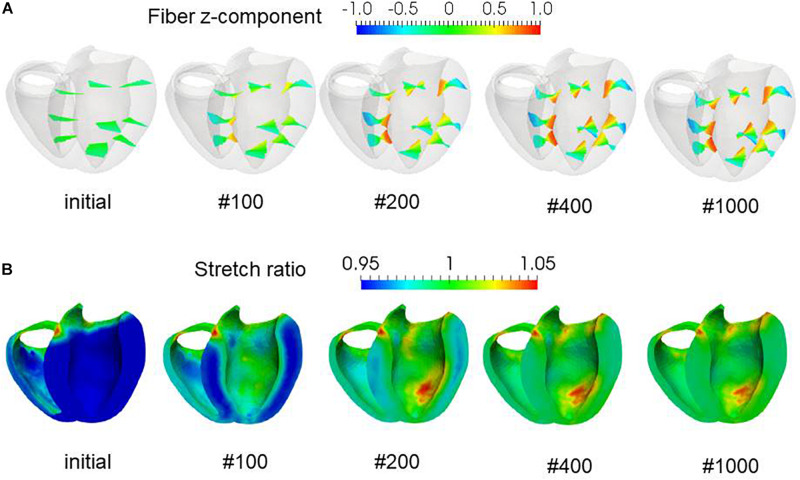
Evolution of fiber helix angles **(A)** and stretch ratio from the end diastolic configuration **(B)** during the reorientation process. The fiber helix angles are shown for the anteroseptal, anterior, and anterolateral zones in **(A)**. The colors indicate the z (longitudinal direction) component of the fiber orientation vector ***f***. The distribution of stretch ratio in the principal fiber direction (1− ∈ _0_) on the infero-endocardial surface and the septal and lateral cross-sections are shown in **(B)**. The numbers below each figure indicate the number of updates. The fiber structure remained stationary after 400 updates.

**FIGURE 4:**
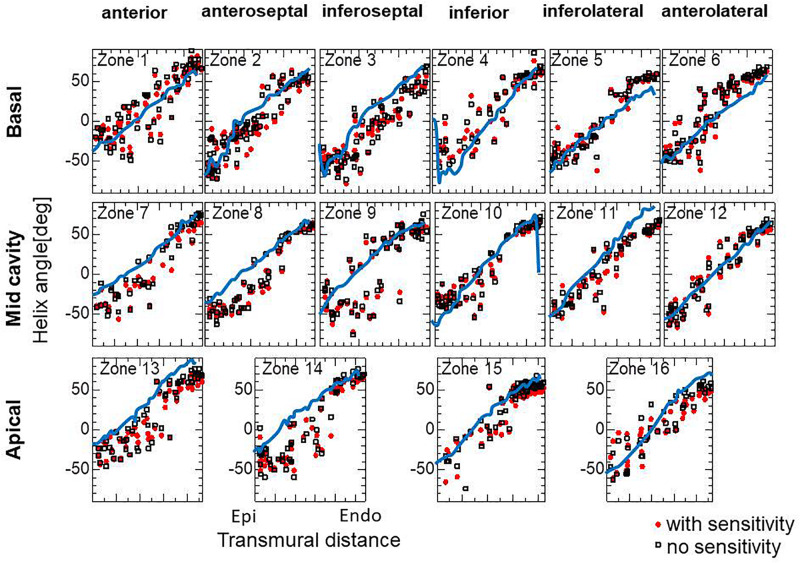
Transmural helical angle arrangement of the optimized fiber structure in American Heart Association segments (The zones 1–16 are shown in Supplementary Material S2). The red dots and black squares were obtained by the reorientation processes with and without the insensitivity mechanism, respectively, and the blue lines were digitized from the diffusion tensor magnetic resonance imaging study reported by Lombaert et al. (2012), which averaged the data obtained from 10 healthy human hearts.

To examine the effectiveness of the reorientation process, we set the initial configuration of the principal unit vector distribution {**f**} of the left ventricle to be nearly circumferential by interpolating the fiber helix angle linearly according to the transmural depth from −10° at the epicardium to + 10° at the endocardium. Such a small twisting in the transmural direction, leaning a little to the physiological fiber structure (−60°at the epicardium to + 60° at the endocardium), was assigned to prevent the reorientation process from heading toward the opposite twisting structure ( + 60° at the epicardium to −60° at the endocardium). The sheet vector distribution {**s**} was set to be perpendicular to the tangential plane at the nearest point on the epicardial surface. The left ventricular intracavitary pressure monotonically increased until it reached a stationary value under the insensitivity mechanism, whereas a gradual decline was observed in the case of the reorientation without the insensitivity mechanism (Figure 2A). Remarkably, the averaged value of Ψ( ∈_0_) (Eq. 5) increased monotonically throughout the iterative process, even in the latter case (Figure 2B). Namely, without the insensitivity mechanism, the reduction in cavity pressure was accompanied by an increase in the averaged active stress. This suggests that maximizing the local stress does not necessarily maximize the intracavitary pressure. The evolution of the fiber helix angle (Figure 3A) shows that the reorientation process effectively stops after the fiber strain has been homogenized (Figure 3B). A detailed comparison of the fiber helix angle with experimental data obtained by a diffusion tensor magnetic resonance imaging study (Lombaert et al., 2012) shows good agreement with the fiber structure of real healthy human ventricles (Figure 4). Note that the helix angles were evaluated with respect to the end diastolic configuration.

### Influence of Inhomogeneity in the Contractility

**FIGURE 5:**
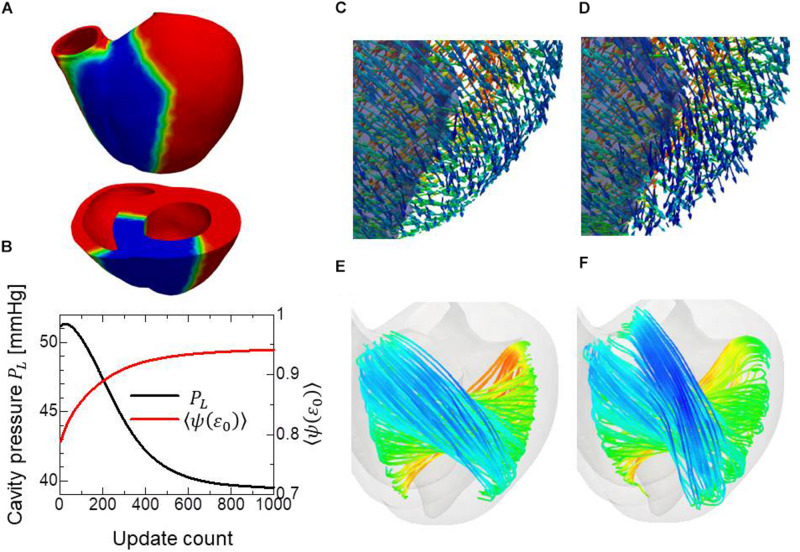
Remodeling of the infarcted ventricle. The remodeling process started from the optimized fiber structure with homogeneous contractility. The region colored blue is the site of infarction, where only 10% of contractility compared to the healthy part was assumed **(A)**. Changes in the left ventricular intracavitary pressure (black line) and the averaged active tension factor (red line) during the remodeling process **(B)**. The distribution of the principal fiber direction ***f*** in the apical anterior region is shown before the remodeling process **(C)** and after the remodeling process with 1000 updates. The infarcted region is shaded **(D)**. The stream lines of the principal fiber direction are shown before the remodeling process **(E)** and after the remodeling process with 1000 updates **(F)**. Here, the colors on the curves indicate the z-component (longitudinal direction) of the principal fiber direction ***f***. The streamlines were constructed by the software PARAVIEW, where the Runge–Kutta 4–5 integrator was adopted.

**FIGURE 6:**
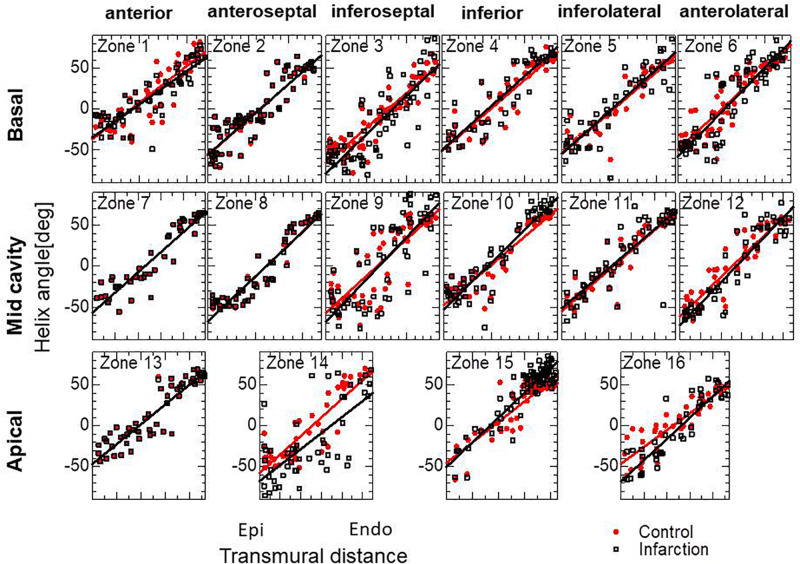
Transmural helical angle arrangement of the optimized fiber structure of the infarcted ventricle model. The black squares dots were obtained by the remodeling processes for the infarcted ventricle model. The red dots indicate the initial arrangement obtained by the optimization for the standard ventricle model. The lines indicate the least-squares approximation of dots with the linear functions.

To examine whether the proposed approach can predict the remodeling after infarction, we applied our reorientation algorithm to the finite element ventricular model with degenerated active stress in specific regions. In this simulation, we started the reorientation from the fiber structure that had been optimized by the procedure described in the previous section to simulate the remodeling from the healthy condition. The region of infarction was as shown in Figure 5A, assuming left anterior descending artery stenosis. In the infarcted region, the reference active tension *T*_*ref*_ was reduced to 10% of the healthy remaining part. In the reorientation process, the left ventricular intracavitary pressure *P*_*L*_ decreases, though the homogeneity of strain is improved (Figure 5B). Near the infarction boundary in the anterior healthy region, the helix angles at the epicardium become sharper, whereas they become gentler at the endocardium (Figures 5C–F). In particular, the fiber integral curves run along the boundary of the infarction, as observed by Sosnovik et al. (2009). In Figure 6, the fiber helix angles are plotted along with those of the initial fiber structure. The distributions have shifted downward slightly at the inferoseptal (Zones 3, 9, and 14) and anterolateral walls (Zones 6, 12, and 16), but are unchanged in the anterior (Zones 1, 7, and 13), which is remote from the site of infarction. This agrees well with the results of experimental studies by Wu et al. (2007, 2011).

### Sensitivity Analysis

**FIGURE 7:**
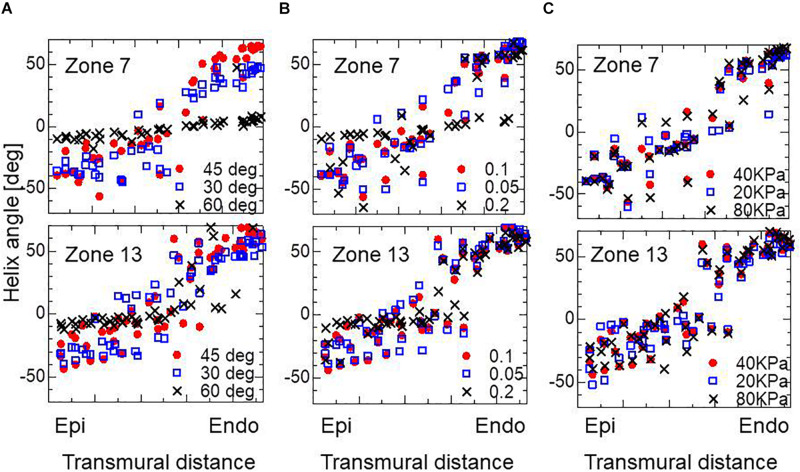
Impacts on the transmural helical angle arrangement of the optimized fiber structure of the variation of dispersion degree of the branching structure θ = 30, 45, and 60° **(A)**, the gradient ∈_*Z*_ = 0.1, 0.05, and 0.2 in defining the stress–strain relationship in the active tension factor **(B)**, and the reference value of the active tension *T*_*ref*_ **=** 20, 40, and 80 kPa **(C)**. The plots for Zone 7: mid cavity-anterior and Zone 13: apical-anterior are shown.

The choice of branching angle θ appears to be essential. In our experience, taking a value of around 45° gives optimal performance. A smaller branching angle enlarged the deviation in helix angle around the middle layer, and made the helix angle at the endocardium slightly smaller, whereas a larger angle diminished the sensitivity to eccentricity in the active tension (Figure 7A). Regarding the choice of the parameter ∈ _*Z*_ in defining the gradient in the stress–strain relationship (Eq. 5), the adoption of a higher value again diminished the sensitivity to eccentricity in the active tension (Figure 7B). In terms of the magnitude of active stress, the impact of the reference value *T*_*ref*_ (Eq. 3) on the optimized fiber structure was small (Figure 7C).

**FIGURE 8:**
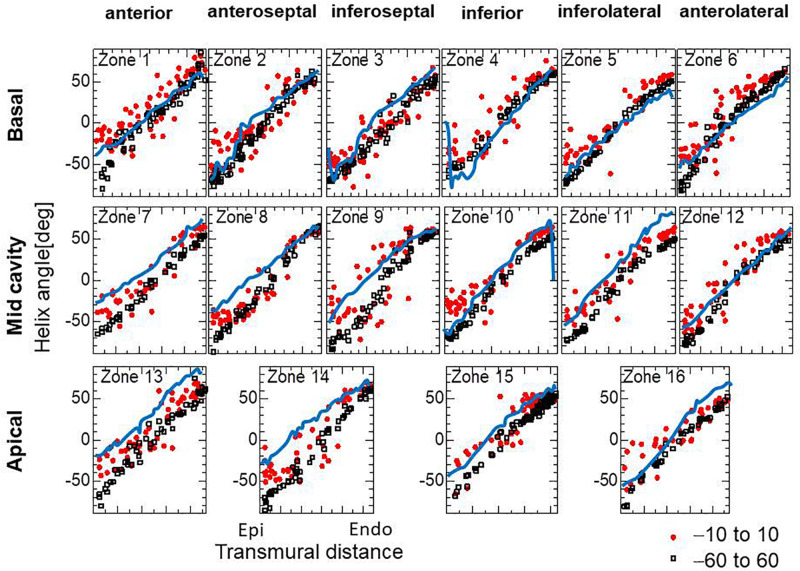
Helical angle arrangement of the optimized fiber structure in American Heart Association segments. The red dots and black squares were obtained by the reorientation processes (1000 updates) starting from the linear transmural distribution from –10° (Epi) to 10° (Endo), and from –60° (Epi) to 60° (Endo), respectively, and the blue lines were digitized from the diffusion tensor magnetic resonance imaging study reported by Lombaert et al. (2012), which averaged the data obtained from 10 healthy human hearts.

**FIGURE 9:**
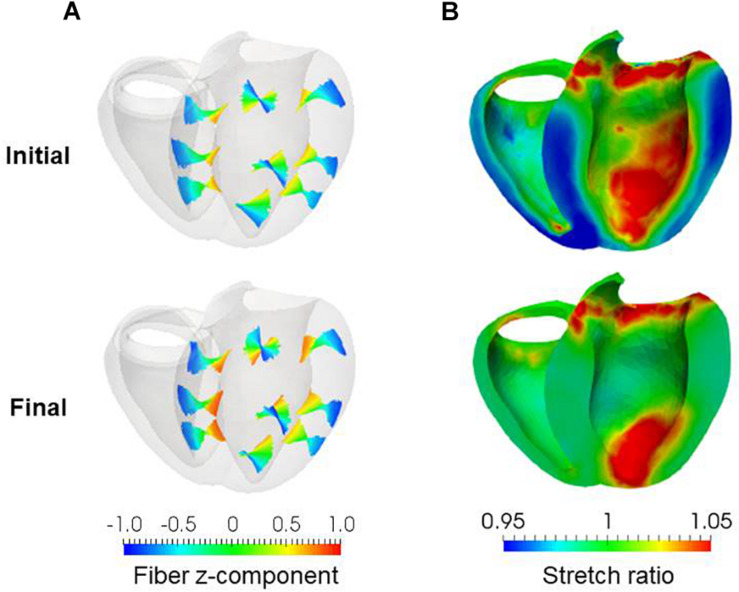
Results of reorientation starting from the linear distribution of fiber helix angle from –60° (Epi) to 60° (Endo). The fiber helix angles are shown for the anteroseptal, anterior, and anterolateral zones in **(A)**. The colors indicate the z (longitudinal direction) component of the fiber orientation vector ***f***. The distribution of stretch ratio in the principal fiber direction (1− ∈_0_) on the infero-endocardial surface and the septal and lateral cross-sections are shown in **(B)**. The upper figures show them for the initial fiber structure, and the lower figures show them for the final structure obtained after 1000 updates.

For the practical use of our approach, it seems more efficient to start the reorientation process from the standard twisting fiber structure. In Figure 8, the optimized fiber structure obtained after 1000 updates starting from an artificial standard structure (linear transmural change from −60° [Epi] to 60° [Endo]) is compared with that starting from a flat distribution (linear transmural change from −10° [Epi] to 10° [Endo]). The resulting structures appear, on average, quite similar for the mid and endocardium layers, although the deviations in the former distributions are much smaller than in the latter. Though the transmural twisting was not markedly different before and after the optimization (Figure 9A), the inhomogeneity of the strain was considerably improved (Figure 9B).

### Reconstruction of Helical Ventricular Myocardial Band

**FIGURE 10:**
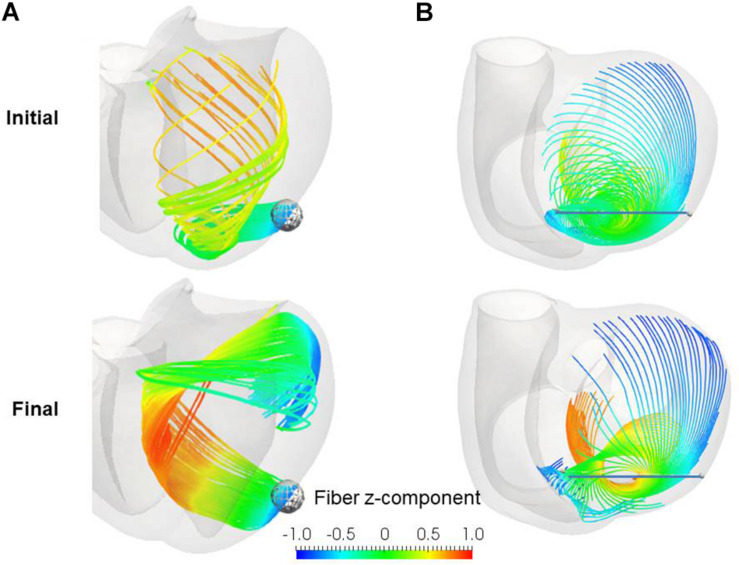
Stream lines before (upper) and after (lower) the reorientation starting from the linear distribution of fiber helix angle from –60° (Epi) to 60° (Endo). **(A)** Stream lines integrated along the principal fiber direction ***f*** from the ball placed at the epicardial apical anterolateral portion. **(B)** Stream lines integrated from the points distributed on the line running through the apex. The colors indicate the z (longitudinal direction) component of the fiber orientation vector ***f***. The streamlines were constructed by the software PARAVIEW, where the Runge–Kutta 4–5 integrator was adopted.

Remarkably, our reorientation process was able to construct the helical ventricular myocardial band running from the apical epicardium to the basal epicardium via the septal endocardium (Figure 10A), as found by Torrent-Guasp (Buckberg et al., 2008). Such stream lines cannot be generated from artificial fiber direction vectors that run perpendicular to the transmural direction everywhere. The spiral structure at the apex (Rushmer et al., 1953; Grant, 1965; Buckberg, 2002) was also accurately reproduced by our reorientation algorithm (Figure 10B). The success of our numerical model in reconstructing these sophisticated structures further validates the applicability of our simple rule in real physiological tissue.

### Pumping Performance

We applied the artificial standard fiber structure (linear transmural change from −60° [Epi] to 60° [Endo]) and the resulting optimized fiber structure to the beating simulation code used in our previous work (Washio et al., 2016). The aim was to confirm that the optimized fiber structure with the simple active stress model in the isovolumetric contraction phase is effective for the whole beating simulation with a physiologically realistic cross-bridge model. In this simulation, the same parameter set of passive material properties as in the reorientation process was applied to eliminate the effect of the laminar structure determined by the sheet direction vector **s**, and the single-directional active stress (Eq. 1) was adopted with the active tension *T*_*f*_ computed by 32 filament pairs in the cross-bridge model for each element. The heart rate was set to 60 beats per minute, and the Ca^2+^-transient generated by the midmyocardial cell model proposed by ten Tusscher and Panfilov (2006) was applied. We used transmural delays determined by the distances from the endocardial surfaces of the left and right ventricles under a transmural condition velocity of 52 cm/s, as measured by Taggart et al. (2000).

**FIGURE 11:**
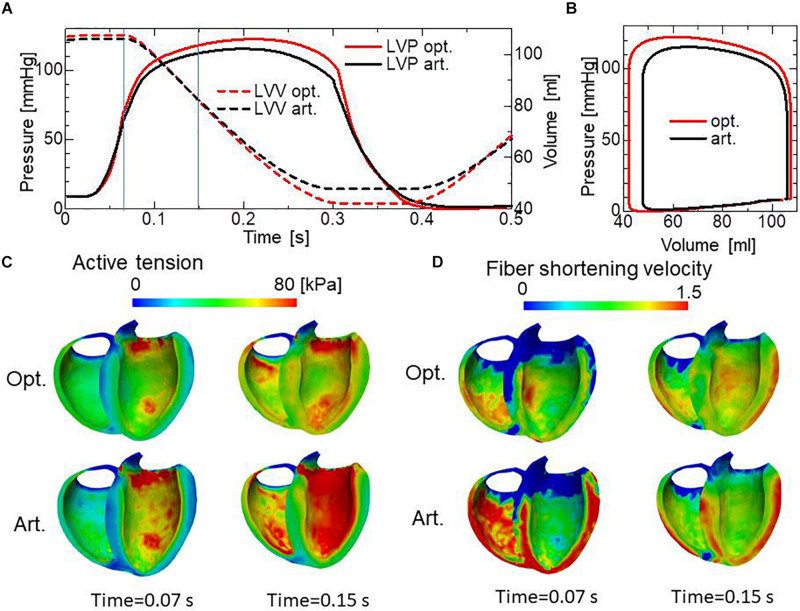
Comparison of pumping performance for the optimized fiber structure and the artificial fiber structure. **(A)** Time courses of left ventricular intracavitary pressures for the optimized fiber structure (red solid line) and the artificial fiber structure (black solid line), and time courses of left ventricular volumes for the optimized fiber structure (red broken line) and the artificial fiber structure (black broken line). **(B)** Pressure–volume loops for the optimized fiber structure (red) and the artificial fiber structure (black). **(C)** Distribution of active tension for the optimized fiber structure (upper) and the artificial fiber structure (lower) at the end of the isovolumetric contraction phase (Time = 0.07 s) and at the middle of the blood ejection phase (Time = 0.15 s). These timings are indicated by the vertical line in **(A)**. **(D)** Distribution of fiber shortening velocity for the optimized fiber structure (upper) and the artificial fiber structure (lower) at the end of the isovolumetric contraction phase (Time = 0.07 s) and at the middle of the blood ejection phase (Time = 0.15 s).

Comparisons of the pumping performance show considerable improvements in the blood ejection [65 vs. 58 ml], rise of blood pressure (122 vs. 115 mmHg) (Figure 11A), and amount of work (1.00 vs. 0.85 J) (Figure 11B) under our reorientation process, which was applied using the simple active stress model in the isovolumetric phase. In comparing the active tension distribution (Figure 11C), the homogenization effects produced by the reorientation mechanism are clearly apparent in both the isovolumetric phase and the blood ejection phase. The correlation in the contours between the active tension (Figure 11C) and the fiber shortening velocity (Figure 11D) indicates the impact of the force–velocity relationship on the contractility. Namely, the active tension is small where the fiber shortening velocity is high.

### Computational Efficiency

**FIGURE 12:**
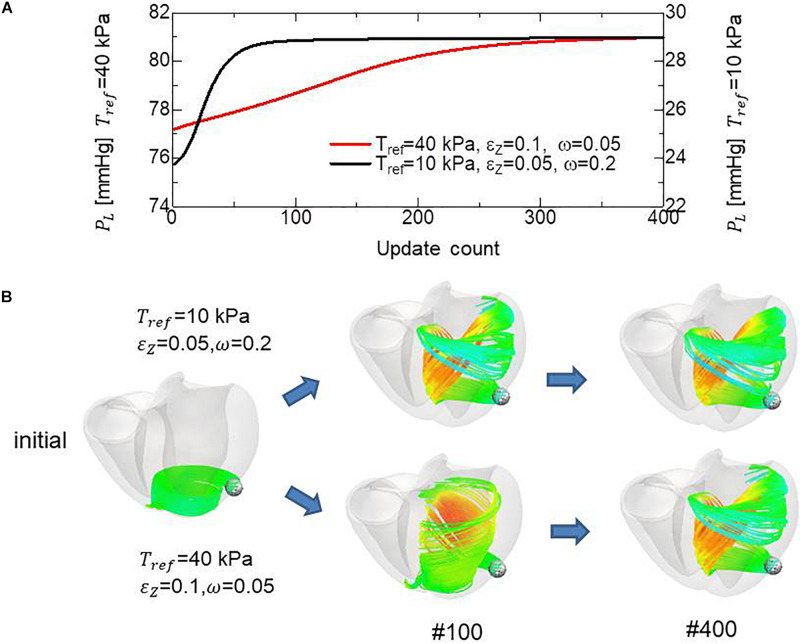
Comparison of remodeling process with the standard parameter set (*T*_*ref*_ = 40 kPa, ∈ _*Z*_ = 0.1, ω = 0.05) and the variant (*T*_*ref*_ = 10 kPa, ∈ _*Z*_ = 0.05, ω = 0.2) for acceleration. **(A)** Transients of the left ventricular intracavitary pressures. **(B)** Evolution of fiber structures are compared by the stream lines starting from the ball placed at the epicardial apical anterolateral portion. The numbers indicate the number of updates. The streamlines were constructed by the software PARAVIEW, where the Runge–Kutta 4–5 integrator was adopted. With the accelerated reorientation process, the increase in cavity pressure and evolution of fiber structure remain almost unchanged after 100 updates.

**FIGURE 13:**
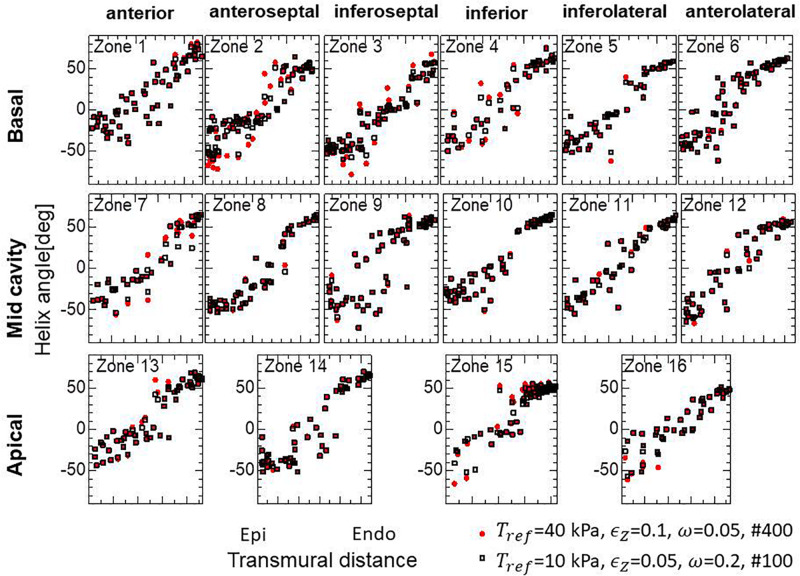
Unchanged transmural helical angle arrangement of the optimized fiber structure in overall left ventricular wall given by the acceleration technique. The red dots and black squares were obtained by 400 updates of the reorientation processes with the standard parameter set (*T*_*ref*_ = 40 kPa, ∈ _*Z*_ = 0.1, ω = 0.05) and by 100 updates with the variant (*T*_*ref*_ = 10 kPa, ∈ _*Z*_ = 0.05, ω = 0.2) for acceleration, respectively. The two distributions are almost the same everywhere in the left ventricle wall.

Because the mechanical equilibrium must be attained for the highly non-linear finite element problem, the reorientation process should be performed carefully to avoid the Newton iterations breaking down. This is the reason for the factor ω = 0.05 in Eq. (16) when modifying the rotation angle $\tilde{\eta }$ to the angle η between **f** and **e**. For practical use in the personalized modeling of fiber structure, it is preferable to reduce the number of updates required to obtain the final structure by using a larger value of ω. Here, we examine whether we can improve the computational efficiency by applying a smaller reference value of the active tension *T*_*ref*_ without degrading the quality of the obtained fiber structure. To maintain computational stability with a larger coefficient value of ω = 0.2, we reduced the reference value *T*_*ref*_ from 40 to 10 kPa (Eq. 3). Because this reduces the strain, we also decreased ∈ _*Z*_ from 0.1 to 0.05 to increase the sensitivity of stress to strain. As shown in Figures 12A,B, the convergence was accelerated by a factor of about four by this strategy. The good agreement in the transmural changes of helix angles everywhere in the left ventricle between the fiber structure after 400 updates with the previous process and after 100 updates with the accelerated process demonstrate the reliability of the acceleration technique (Figure 13). With this acceleration, the computation time was reduced to 0.4 h (100 updates) from 1.6 h (400 updates). This result clearly indicates that the optimized fiber structure is insensitive to the magnitude of the contractile tension, suggesting that large deformation analysis is not always necessary for predicting the fiber orientation.

## Discussion

In this study, a simple stress–strain relationship (Eq. 5) under static equilibrium was used for myofiber reorientation of the biventricular finite element model. This relationship was introduced as a replacement for the force–velocity relationship in the whole dynamical systole. The simplification provided an insight into the reorientation mechanism using the microscopic branching fiber architecture. Namely, the force–velocity relationship implies less active tension for faster shortening. This corresponds to the property of the stress–strain relationship that represents lower stress with greater shortening strain. Our approach using the association of the two relationships under the dynamic and static conditions was verified by the unaffected fiber structures for the magnitudes of active tension (Figures 7C, 12, 13) and the beating ventricle simulation results (Figure 11).

While the reorientation mechanism worked well in terms of improving the pumping performance when the muscle contractility was homogeneous (Figure 3), it was also shown that the same mechanism may negatively affect the pumping performance for the ventricles that contain regions severely damaged by infarction. In our numerical model, the mechanism modified the fiber structure in the healthy region near the site of infarction such that the fiber stream lines run along that boundary (Figures 5D,F). As a result, the connections between the healthy region and the site of infarction are weakened, which results in the region around the boundary becoming elongated in the cross-fiber direction and a decrease in intracavitary pressure (Figure 5B).

The successful reconstruction of the helical ventricular myocardial band of Torrent-Guasp’s model and the apical spiral structure of Rushmer’s model indicate that these interesting structures should be inevitable results of the simple reorientation mechanism, regardless of their roles in pumping. Our analysis suggests that these structures make the myocytes support each other along their longitudinal direction, thus minimizing the overall fiber shortening strains.

Our macroscopic multidirectional active tensor was shown to be useful for predicting the fiber structure, and also for understanding the reorientation mechanism using the microscopic branching structure. However, we must be careful in applying this tensor to beating heart dynamical simulations, because such multidirectional stress degrades the pumping performance, with the degradation becoming worse as the dispersion angle θ in Eq. 7 increases. Actually, in our model with the common reference active tension *T*_*ref*_ = 40 kPa, the left ventricular intracavitary pressures for the optimized structure were 89.7 mmHg and 79.0 mmHg, respectively, for θ = 30° and θ = 45°. Because we assumed a nearly incompressible property for the muscle, the active tension in one direction imposes expansions in orthogonal directions. Therefore, the active tensions in the branching directions interfere with each other. However, it is not known whether the real microscopic branching architecture has the same disadvantage, because our macroscopic model may be too simple to reproduce the sophisticated characteristics of the branching architecture. Another point of concern is the disorder of the fiber helix angle—it is not certain whether the disorder was generated by some characteristic errors of finite element discretization. Apparently, the disorder is not improved after being generated during the reorientation process. Indeed, when the initial structure was closer to the optimal structure, the disorder became considerably smaller, even after the same number of iterations (Figure 9A). Note that the intracavitary pressure also increased from 79.0 to 82.3 mmHg when the initial structure was changed. Thus, the question arises as to whether there is some mechanism for diminishing the disorder. The numerical smoothing of disorder is not so simple, because **f** and −**f** are equivalent in terms of defining the active stress tensor, but not for smoothing the distribution of **f**.

Our main goal of this study is to construct a tool to predict the fiber structure for individual heart disease patients, and use the obtained data as the input for computer simulations to optimize the treatments. Though the validation for the healthy ventricle in Figure 4 and the infarcted ventricle in Figures 5, 6 show the potential of our algorithm as such a tool, we must further validate it with animal models of regional infarct, pressure overload, and volume overload. Because our algorithm is based on the static mechanical equilibrium in the isovolumetric systole, it can’t be directly applied to cases of dyssynchrony or diastolic dysfunction. In case of dyssynchrony, some regional weighting to the active stress according to the excitation timing (smaller weight to latterly excited region) may be effective. In case of diastolic dysfunction in which smooth relaxation of sarcomeres is hindered due to residual cross-bridges of actomyosin complex, our algorithm may be valid by properly reproducing the end diastolic configuration as the starting point of our optimization. Further investigations by comparisons with experimental facts are needed for these issues.

In this study, we did not consider the role of the laminar structure indicated by the sheet orientation vector **s**. We have tested the reorientation process with non-symmetric weights such as *w*_0_ = 1/2,*w*_1_ = *w*_5_ = 1/8,*w*_2_ = *w*_4_ = *w*_6_ = *w*_8_ = 1/16,*w*_3_ = *w*_7_ = 0 in Eq. (7), and updated the frame {**f**,**s**,**n**} using the two eigenvectors associated with the first- and second-largest eigenvalues of the tensor: $\text{D}=\sum_{i=0}^{8}w_{i}\,\psi_{i}\,\left(\in_{i}\right)\,{\text{f}}_{i}⊗{\text{f}}_{i}$. However, the intracavity pressure decreased by 19 mmHg compared with the fiber structure obtained by the reorientation process using symmetric weights, and the disorder in helix angles increased considerably. Therefore, further studies are needed to identify the role of the laminar structure, including its impact on the passive properties.

In the ventricular model used in this study, the papillary muscles were neglected. Thus, the mechanical loads imposed by pulling forces via chordae tendineae were ignored. Because the total pulling force via chordae tendineae is about the same as the product of valve area and intracavitary pressure, it may give considerable impact on the fiber architecture around the roots of the papillary muscles. In fact, the gaps at the epicardial region in zones 9 and 14 around the apical and mid septal segments (see also Supplementary Material S2) between the numerical results and the experimental data in Figure 4 may be caused by the removal of a right ventricular papillary muscle attached to the septal inner wall. The inclusion of papillary muscles in the numerical ventricle model along with their appropriate mechanical treatment is our future work.

## Data Availability Statement

The datasets generated for this study are available on request to the corresponding author.

## Author Contributions

SS and TH designed the project. TW designed and conceived the numerical algorithm of reorientation. TW and JO constructed the simulation code and the input data. TW wrote the manuscript with inputs from SS and TH.

## Conflict of Interest

TW, SS, JO, and TH were employed by the company UT-Heart Inc.
