# Supplementary material for: Using Systolic Local Mechanical Load to Predict Fiber Orientation in Ventricles
Source: Front Physiol. 2020 Jun 9;11:467. doi: 10.3389/fphys.2020.00467 (PMC7295989; doi:10.3389/fphys.2020.00467)
Supplement: Supplementary file 1 [file Data_Sheet_1.PDF]

Supplementary Material

Using systolic local mechanical load to predict fiber orientation in ventricles

Takumi Washio[1,2*], Seiryo Sugiura[1], Jun-ichi Okada [1,2], Toshiaki Hisada[1]

[1]UT-Heart Inc., Kashiwanoha Campus Satellite, 178-4-4 Wakashiba, Kashiwa, Chiba 277-0871, Japan

[2]Future Center Initiative, University of Tokyo, Kashiwanoha Campus Satellite, 178-4-4 Wakashiba, Kashiwa, Chiba 277-0871, Japan

*** Correspondence:**
Takumi Washio
washio@ut-heart.com

S1 Mathematical notations and symbols

Let $\{\boldsymbol{e}_1, \boldsymbol{e}_2, \boldsymbol{e}_3\}$ be an orthonormal basis of the three-dimensional Euclidean space. For vectors $\boldsymbol{a} = \sum_{i=1}^{3} a_i \boldsymbol{e}_i$ and $= \sum_{i=1}^{3} b_i \boldsymbol{e}_i$, the dot product is defined as

$$\boldsymbol{a} \cdot \boldsymbol{b} = \sum_{i,j=1}^{3} a_i b_i , \tag{S1}$$

and the tensor product is defined as

$$\boldsymbol{a} \otimes \boldsymbol{b} = \sum_{i,j=1}^{3} a_i a_j \boldsymbol{e}_i \otimes \boldsymbol{e}_j . \tag{S2}$$

The tensor product is regarded as the linear mapping in the Euclidean space:

$$\boldsymbol{a} \otimes \boldsymbol{b} \cdot \boldsymbol{c} = (\boldsymbol{b} \cdot \boldsymbol{c})\boldsymbol{a}, \forall \mathbf{c} . \tag{S3}$$

The Euclidean norm is given by $\|\boldsymbol{a}\| = (\boldsymbol{a} \cdot \boldsymbol{a})^{\mathbf{1/2}}$**.** The scalar product of two tensors $\boldsymbol{A} = \sum_{i,j=1}^{3} A_{ij}\, \boldsymbol{e}_i \otimes \boldsymbol{e}_j$ and $\boldsymbol{B} = \sum_{i,j=1}^{3} B_{ij}\, \boldsymbol{e}_i \otimes \boldsymbol{e}_j$ is defined as

$$\boldsymbol{A} : \boldsymbol{B} = \sum_{i,j=1}^{3} A_{ij} B_{ij} . \tag{S4}$$

Under the above definition, the active stress tensor is derived by considering the virtual work as follows. Assume that the current position of each material point $\boldsymbol{X} \in \Omega$ in the reference configuration

is represented as $\boldsymbol{x} = \boldsymbol{x}(\boldsymbol{X})$. The virtual work for the infinitesimal increment $\boldsymbol{\delta x}$ of the current position $\boldsymbol{x}$ under the active tension T_f along the fiber orientation $\boldsymbol{f}$ on the infinitesimal volume $d\Omega$ in the reference space is given by

$$\delta W_{act} = T_f \delta \|\boldsymbol{Ff}\| d\Omega = T_f \delta \left\{ (\boldsymbol{Ff} \cdot \boldsymbol{Ff})^{\frac{1}{2}} \right\} d\Omega = \frac{T_f}{\|\boldsymbol{Ff}\|} \frac{1}{2} \delta(\boldsymbol{Ff} \cdot \boldsymbol{Ff}) d\Omega = \frac{T_f}{\|\boldsymbol{Ff}\|} \frac{1}{2} \delta(\boldsymbol{f} \otimes \boldsymbol{f} : \boldsymbol{F}^T \boldsymbol{F}) d\Omega$$
$$= \frac{T_f}{\|\boldsymbol{Ff}\|} \boldsymbol{f} \otimes \boldsymbol{f} : \boldsymbol{\delta E} d\Omega, \qquad \text{(S5)}$$

where $\boldsymbol{F} = \partial \boldsymbol{x} / \partial \boldsymbol{X}$ is the deformation gradient tensor, $\boldsymbol{\delta E} = \frac{1}{2}(\boldsymbol{\delta F}^T \boldsymbol{F} + \boldsymbol{F}^T \boldsymbol{\delta F})$ is the infinitesimal increment of the Green–Lagrange strain tensor $\boldsymbol{E} = \frac{1}{2}(\boldsymbol{F}^T \boldsymbol{F} - \boldsymbol{I})$.

As a result, with the second Piola–Kirchhoff active stress tensor represented as

$$\boldsymbol{S}_{\mathrm{act}} = \frac{T_f}{\|\boldsymbol{Ff}\|} \boldsymbol{f} \otimes \boldsymbol{f}, \qquad \text{(S6)}$$

the virtual work is given by

$$\delta W_{act} = \boldsymbol{\delta E} : \boldsymbol{S}_{\mathrm{act}} d\Omega. \qquad \text{(S7)}$$

From the definition of second Piola–Kirchhoff active stress tensor, the traction force in the current configuration acting on an area element $\boldsymbol{N} dA$ is given by

$$\boldsymbol{dt} = \boldsymbol{F} \boldsymbol{S}_{act} \boldsymbol{N} dA, \qquad \text{(S8)}$$

where the normal vector $\boldsymbol{N}$ points outward from the area dA. As shown in Figure S2, let da be the infinitesimal area in the current configuration corresponding to dA, and $\boldsymbol{n}$ be its outward normal vector. Then, from Nanson's formula: $\boldsymbol{n} da = det(\boldsymbol{F}) \boldsymbol{F}^{-T} \boldsymbol{N} dA$ and Equations (S6) and (S8), the traction force is rewritten as

$$\boldsymbol{dt} = \frac{T_f}{\|\boldsymbol{Ff}\|} \boldsymbol{F}(\boldsymbol{f} \otimes \boldsymbol{f}) \frac{1}{det(\boldsymbol{F})} \boldsymbol{F}^T \boldsymbol{n} da = \frac{T_f}{(det(\boldsymbol{F}) / \|\boldsymbol{Ff}\|)} \left(\frac{\boldsymbol{Ff}}{\|\boldsymbol{Ff}\|} \otimes \frac{\boldsymbol{Ff}}{\|\boldsymbol{Ff}\|} \right) \boldsymbol{n} da. \qquad \text{(S9)}$$

Thus, with the corresponding Cauchy stress tensor:

$$\boldsymbol{T}_{act} = \frac{T_f}{(det(\boldsymbol{F}) / \|\boldsymbol{Ff}\|)} \left(\frac{\boldsymbol{Ff}}{\|\boldsymbol{Ff}\|} \otimes \frac{\boldsymbol{Ff}}{\|\boldsymbol{Ff}\|} \right), \qquad \text{(S10)}$$

the traction force is given by

$$\boldsymbol{dt} = \boldsymbol{T} \boldsymbol{n} da. \qquad \text{(S11)}$$

Note that the scaler factor $T_f / (det(\boldsymbol{F}) / \|\boldsymbol{Ff}\|)$ in the right hand side in Equation (S10) is nothing but the active tension with respect to the current configuration.

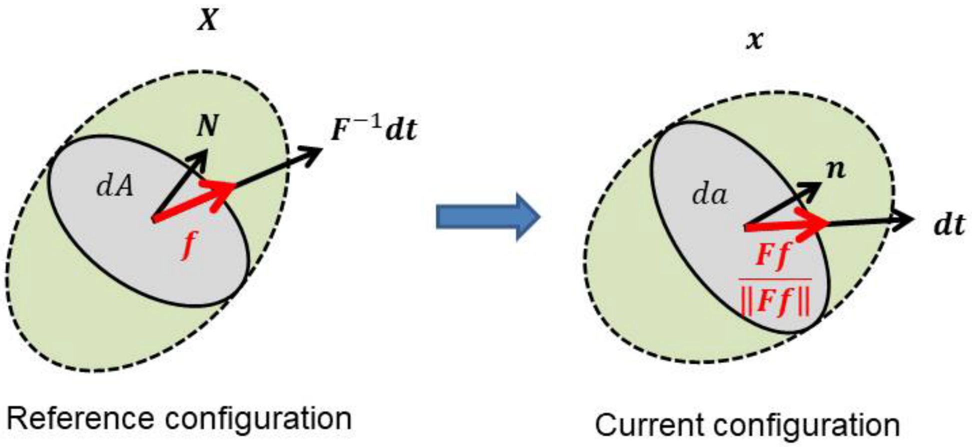

Figure S1 Infinitesimal areas and their normal vectors in the reference (left) and current (right) configurations. Here, $da = \{\boldsymbol{x}(\boldsymbol{X}) : \boldsymbol{X} \in dA\}$, the normal vector $\boldsymbol{N}$ points outward from the area dA, and $\boldsymbol{n}$ points outward from the area da. $\boldsymbol{f}$ is the fiber orientation normal vector in the reference configuration.

S2 American Heart Association segments

Figure S2 shows the change in optimized fiber orientation obtained by the reorientation processes with the insensitivity mechanism at each of 17 American Heart Association segments in the left ventricle.

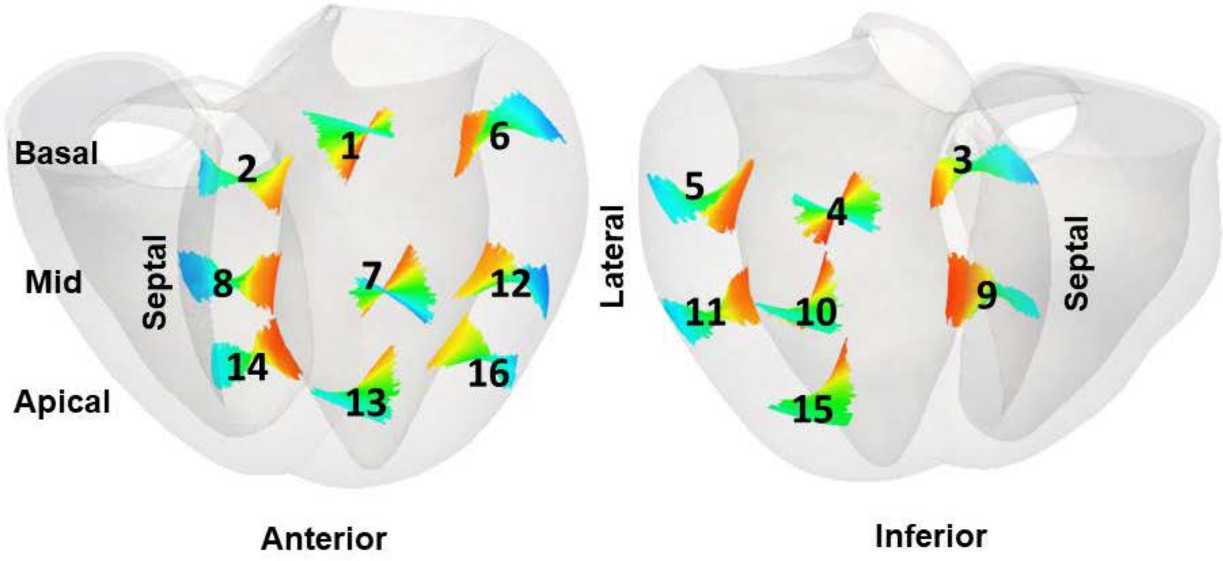

Figure S2. The fiber orientation obtained by the reorientation processes with the insensitivity mechanism at each of 17 American Heart Association segments. The anterior (left) and inferior (right) views are presented.
